# Clinical Significance of Prognostic Nutritional Index in Patients Who Underwent Palliative Surgery for Spine Metastasis

**DOI:** 10.3390/jcm14124372

**Published:** 2025-06-19

**Authors:** Young-Hoon Kim, Kee-Yong Ha, Hyung-Youl Park, Kihyun Kwon, Yunseong Kim, Hyun W. Bae, Sang-Il Kim

**Affiliations:** 1 Department of Orthopedic Surgery, Seoul St. Mary’s Hospital, College of Medicine, The Catholic University of Korea, Seoul 06591, Republic of Korea; boscoa@catholic.ac.kr (Y.-H.K.); kyh@catholic.ac.kr (K.-Y.H.); 899835@naver.com (K.K.); cinar@naver.com (Y.K.); 2 Department of Orthopedic Surgery, Eunpyeong St. Mary’s Hospital, College of Medicine, The Catholic University of Korea, Seoul 03312, Republic of Korea; matrixbest@naver.com; 3 Department of Orthopedic Surgery, Cedars-Sinai Medical Center, Los Angeles, CA 90048, USA; baemd@me.com

**Keywords:** spine metastasis, palliative surgery, nutrition, prognostic nutritional index, ambulatory function, survival

## Abstract

**Background/Objectives**: Malnutrition is common in patients with metastatic spine tumors (MSTs) and may adversely affect surgical outcomes. The Prognostic Nutritional Index (PNI) reflects both nutritional and immune status, but its role in palliative MST surgery is not well defined. The aim of this study was to investigate the association between preoperative the PNI and postoperative outcomes, including functional recovery and survival, in patients undergoing palliative surgery for MSTs. **Methods**: A brief description of the main methods or treatments applied. This can include any relevant preregistration or specimen information. **Results**: Patients with a higher PNI (≥42.8) demonstrated significantly better postoperative ambulation and longer overall survival compared to those with a lower PNI (<42.8). The higher PNI group showed earlier ambulation (*p* = 0.017) and longer median survival (30.7 vs. 7.0 months; *p* = 0.002). Multivariate analysis confirmed that a PNI ≥ 42.8 was an independent predictor of early ambulation (HR = 1.516; 95% CI: 1.010–2.277; *p* = 0.045) and prolonged survival (HR = 0.955; 95% CI: 0.927–0.985; *p* = 0.003). No significant association was found between the PNI and postoperative infections. **Conclusions**: The PNI is a simple and effective predictor of postoperative functional recovery and survival in patients undergoing palliative surgery for MSTs. Its routine preoperative assessment may help stratify surgical risk, guide nutritional interventions, and optimize clinical outcomes in this vulnerable population.

## 1. Introduction

A recent epidemiological study reported that approximately 23 million people had cancer in 2019, which was 2.3 times than in 1990 [1]. Bone is the most common site for the metastases of many cancers. Most cancers often metastasize to the axial skeleton, with the spine being the most frequent site of bone metastasis, accounting for 87% [2,3]. Although not all spine metastases will have clinical manifestations, symptomatic spine metastasis can cause pain, neurological deficits, and even the loss of ambulatory function, leading to increased mortality and decreased quality of life [4,5]. The treatment goal for metastatic spine tumors (MSTs) is predominantly palliative to reduce pain and to preserve or improve neurologic status [4,5]. It has been reported that the surgical treatment for MSTs has increased over the past few decades [6,7]. Surgical treatment can influence subsequent additional cancer treatment and even overall survival [8,9]. Although surgical techniques have improved, postoperative complications are quite common after surgical treatment for MSTs because cancer patients are usually frail and malnourished with medical comorbidities and poor functional status [10].

Malnutrition is a common disorder in cancer patients, accounting for 40%, especially in those with advanced stages [11,12]. Although the pathophysiology of malnutrition is complex, it is mainly a consequence of inadequate food intake caused by cancer itself or side effects during anticancer treatment in cancer patients [13]. Besides starvation-type malnutrition, there is another type of malnutrition called inflammation-type malnutrition, in which systemic inflammation increases due to metabolic derangement. Both types of malnutrition can put patients at greater risk of poorer adverse events, such as increased morbidity, complications, length of stay, and even mortality [14,15].

Various scoring tools are available to assess nutritional status such as the Mini Nutritional Assessment, Prognostic Nutritional Index (PNI), and Nutritional Risk Index (NRI). The PNI is calculated using serum albumin and lymphocyte count, which make it easier to use than other metrics [16]. The serum albumin level is a widely used indicator for nutritional status and disease status of cancer patients [17]. Lymphocyte count is reflective of inflammatory status. Elevated systemic inflammation in cancer patients is associated with poor prognosis and the development of inflammation-type malnutrition [18]. Recent studies have found that the PNI has significant prognostic value in cancer treatment [19,20,21,22].

Despite growing evidence supporting the utility of the PNI in oncology, its role in the specific context of palliative surgery for MSTs remains underexplored. Given that functional recovery and quality of life are primary goals in this population, identifying reliable preoperative prognostic markers is essential. Moreover, considering the high prevalence of malnutrition in patients with MSTs, preoperative nutritional assessment using the PNI could help stratify surgical risk and guide perioperative management. Therefore, this study aimed to investigate the association between the preoperative PNI and postoperative outcomes, including functional recovery and overall survival, in patients undergoing palliative surgery for MSTs. By elucidating this relationship, we hope to provide evidence that supports the integration of nutritional assessment into preoperative risk stratification and clinical decision making in this vulnerable patient population.

## 2. Materials and Methods

### 2.1. Patient Selection

This study was approved by the Institutional Review Board of our institute (approval No. KC24RISI0293, approval date 7 May 2024). This retrospective study used prospectively collected data for all patients who underwent spine surgery for MSTs in a tertiary single institute from January 2017 to June 2021. During the study period, 195 patients underwent spine surgery for MSTs in our institute. Inclusion criteria were as follows: age of more than 18 years, palliative debulking surgery, and preoperative preservation of independent ambulatory function. If the surgery was performed to relieve preoperative symptoms caused by a metastatic spine tumor but was not intended as a curative excision, the authors defined it as “palliative debulking surgery.” We adapted the Nurick grading system to evaluate ambulatory function [23]. If the patient showed a Nurick grade 4 or higher (able to walk with an aid), we determined that the patient was ambulatory. Exclusion criteria were as follows: preoperative Nurick grade 5 (chair-bound or bedridden), curative surgery, any preoperative nutritional interventions, revision surgery at the index level, lack of data, or follow-up period of less than 30 days after surgery.

### 2.2. Data Collection

We recorded clinical and surgical data. Patient data included age, sex, and body mass index (BMI). Clinical data included American Society of Anesthesiologists (ASA) classification, Eastern Cooperative Oncology Group (ECOG) performance status, primary cancer, history of oncological treatment (chemotherapy or radiotherapy), and modified Charlson Comorbidity Index (mCCI) [24]. Because all patients had cancer, the mCCI was calculated with the exclusion of 6 points for cancer. Surgery-related data included the length of operated segments, estimated blood loss (EBL), use of instrumentation, and need for intensive care unit (ICU). PNI was calculated with the following formula: 10 × serum albumin (g/dL) + 0.005 × total lymphocyte count (/mm^3^) [16]. Currently, there is no consensus on the normal range of PNI. Thus, we divided patients into two groups and compared them based on the mean PNI value of our cohort. Perioperative complications including surgical site infection and other infectious conditions (e.g., pneumonia, cellulitis) were investigated. Surgical site infection (SSI) (either superficial or deep) was diagnosed based on the definition of the Centers for Disease Control and Prevention [25]. Postoperative times to start ambulation and postoperative survival were reviewed.

### 2.3. Statistical Analysis

All statistical analyses were performed using SPSS software (IBM SPSS Statistics for Windows, v24; IBM Corp., Sydney NSW, Australia). Continuous variables are expressed as mean and standard deviation, and they were analyzed with unpaired *t*-test. Categorical variables are expressed as absolute numbers and percentages, and they were analyzed with chi-square test or Fisher’s exact test as appropriate. Survivorship was subjected to Kaplan–Meier analysis. Log-rank test was used to compare outcomes between groups. Cox proportional hazard models were used to analyze the correlation between PNI and postoperative ambulatory function or survival. Receiver operating characteristic (ROC) analysis was performed for postoperative ambulatory function and overall survival. For all analyses, *p* values of less than 0.05 were considered significant.

## 3. Results

### 3.1. Preoperative and Operative Details

**Figure 1:**
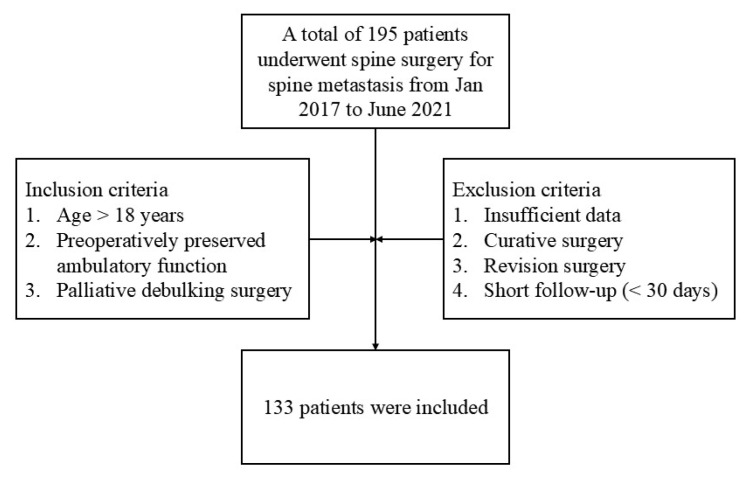
Flow chart for inclusion and exclusion.

Among 195 patients who underwent surgery during the study period, a total of 133 patients met the criteria and were included in this study (Figure 1). Baseline demographic and oncological details of these 133 patients are shown in Table 1. Of these patients, 78 (58.6%) were male. The mean age was 61.1 years for the 133 patients included in this study. The preoperative mean BMI was 23.0 ± 3.3 kg/m^2^, the mean mCCI was 7.1 ± 1.3, and the mean PNI was 42.8 ± 7.5. The mean postoperative follow-up period was 15.8 ± 14.1 months. Multiple myeloma (*n* = 35) and lung cancer (*n* = 34) were the most common primary cancers, followed by breast cancer (*n* = 13). Perioperative data are summarized in Table 2. A total of 119 patients underwent instrumented surgery, and the remaining 14 patients underwent decompression surgery without instrumentation. The mean number of operated levels was 3.8 ± 1.7 for instrumented surgery and 1.9 ± 1.0 for decompression only. Mean perioperative blood loss was 906.6 ± 815.9 mL, and postoperative ICU care was needed for 44 patients (33.1%). Mean values of serum albumin level and lymphocyte count were 3.7 ± 0.6 g/dL and 1215 ± 590/mm^3^, respectively. The mean PNI was 42.8 ± 7.5.

### 3.2. Relationship Between PNI and Infection

SSI was identified in 10 patients (7.5%). Comparing patients with and without SSI, there was no significant difference in the PNI between the two groups (39.5 ± 6.2 vs. 43.1 ± 7.6, *p* = 0.148). Infectious conditions other than SSI occurred in 13 patients (9.8%). Comparing patients with and without infectious conditions, there was no significant difference in the PNI between the two groups either (41.1 ± 11.5 vs. 43.0 ± 7.0, *p* = 0.568) (Table 3).

### 3.3. Relationship Between PNI and Ambulatory Function

**Figure 2:**
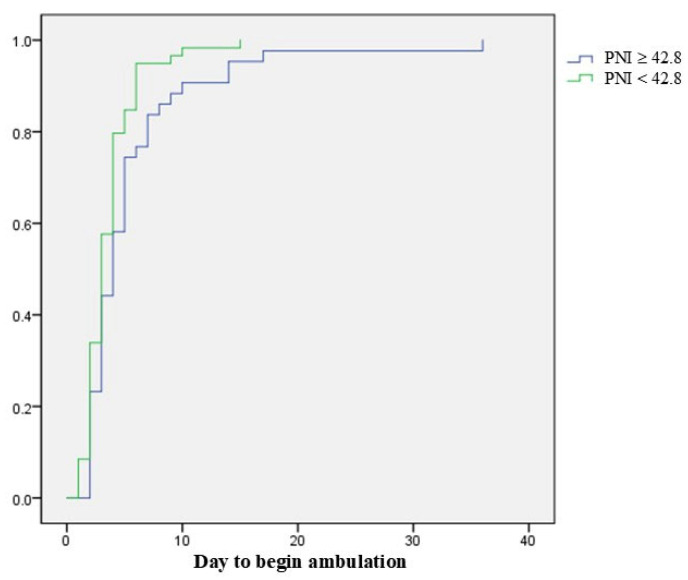
Log-rank test shows that patients with a PNI higher than 42.8 show earlier ambulatory function after surgery than those with a PNI less than 42.8 (*p* = 0.017).

**Figure 3:**
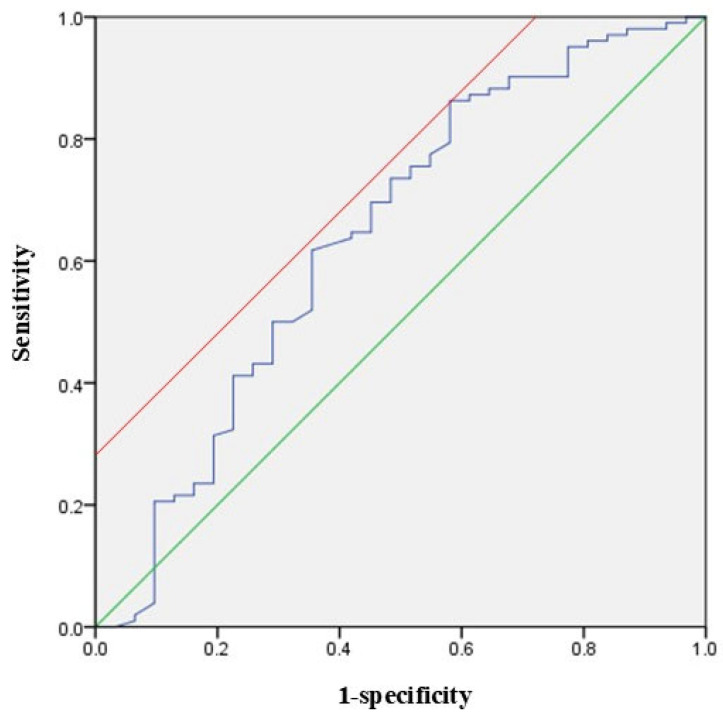
ROC curve for ambulatory function at postoperative 1 month. A green line indicates the diagonal reference line and a red line indicates the a tangent representing the Youden index for the optimal cutoff point.

At postoperative 1 month, 102 patients (77.3%) showed ambulatory function. They had a higher preoperative PNI than 30 patients with the loss of ambulatory function (43.7 ± 6.8 vs. 39.6 ± 9.1, *p* = 0.008). We performed a sub-analysis for 102 patients with postoperative ambulatory function. When analyzing two groups divided based on the mean value of the PNI (42.8), patients with a higher PNI (≥42.8) showed earlier ambulatory function after surgery than those with a lower PNI on the log-rank test (*p* = 0.017) (Figure 2). In the multivariate Cox biohazard model, a PNI greater than 42.8 was a significantly positive factor for earlier postoperative ambulation (HR = 1.516; 95% CI = 1.010–2.277; *p* = 0.045). The ROC curve for ambulatory function at postoperative 1 month is shown in Figure 3. Area under the curve (AUC) was 0.642, and the optimal cut-off value of the PNI was 36.1 (sensitivity = 0.863, specificity = 0.419).

### 3.4. PNI and Postoperative Survival

**Figure 4:**
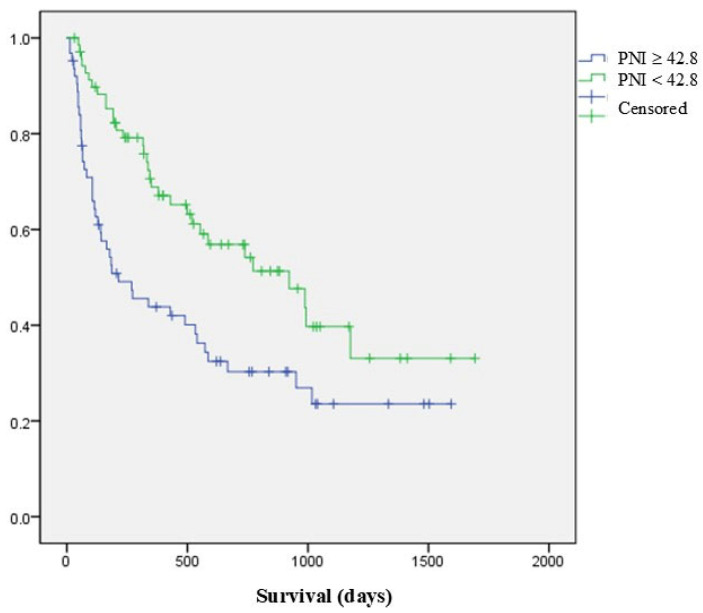
Log-rank test shows that patients with a PNI higher than 42.8 have longer overall survival (median of 30.7 months; 95% CI for 15.9–45.5 months) than those with a PNI lower than 42.8 (median of 7 months; 95% CI for 1.1–13.2 months; *p* = 0.002).

**Figure 5:**
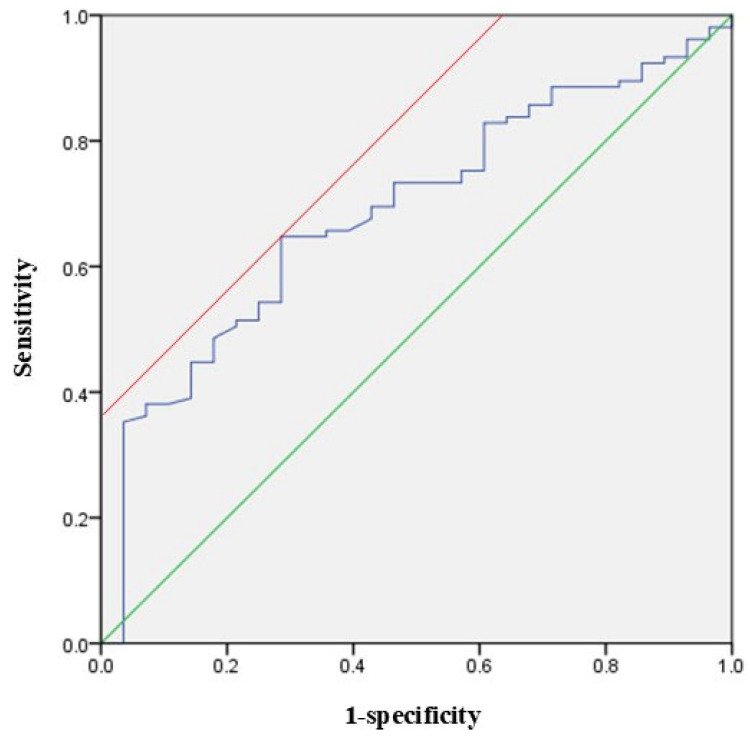
ROC curve for overall survival at postoperative 1 month. A green line indicates the diagonal reference line and a red line indicates the a tangent representing the Youden index for the optimal cutoff point.

Median overall survival of all patients was 540 days. Mortality rates at 3 months and 12 months were 81.6% and 57.1%, respectively. A log-rank test demonstrated that patients with a PNI higher than 42.8 had longer median overall survival (30.7 months; 95% CI 15.9–45.5 months) than those with a PNI lower than 42.8 (7 months; 95% CI 1.1–13.2 months, *p* = 0.002) (Figure 4). A lower PNI (< 42.8) was associated with a significantly lower median overall survival (HR = 0.955; 95% CI = 0.927–0.985; *p* = 0.003). The ROC curve for overall survival at postoperative 3 months is shown in Figure 5. The area under the curve (AUC) was 0.688, and the optimal cut-off value of the PNI was 42.0 (sensitivity = 0.648, specificity = 0.714).

## 4. Discussion

The PNI, which consists of nutritional (serum albumin level) and immunological (lymphocyte count) indices, is widely used as a predictive indicator of postoperative morbidities and mortality in cancer patients [19,20,21,22]. This study investigated the prognostic significance of the preoperative PNI in patients undergoing palliative surgery for MSTs. Our findings demonstrate that a lower PNI is significantly associated with delayed postoperative ambulation and decreased overall survival. Although the PNI was not significantly associated with postoperative infections, its correlations with functional and survival outcomes underscore its clinical value. The PNI, derived from serum albumin and lymphocyte count, reflects both nutritional status and systemic immune function. Patients with a PNI ≥ 42.8 were more likely to achieve early ambulation. They had a median overall survival of 30.7 months, higher than a median overall survival of 7.0 months for patients with a lower PNI. These results are clinically meaningful, particularly in the context of palliative surgery, where functional recovery and quality of life are primary goals.

The possible relationship between a low PNI and impaired ambulatory recovery likely reflects underlying sarcopenia. Although we did not directly measure skeletal muscle mass, sarcopenia and malnutrition often coexist in advanced cancer, particularly under chronic systemic inflammation [26,27]. Our recent study has found that both the PNI and the psoas muscle index are independent predictors of postoperative recovery and complication risk in MST surgery [28]. Similarly, Ushiku et al. have demonstrated that skeletal muscle mass assessed by total psoas area (TPA)/vertebral body area can predict short-term postoperative function [29]. These studies highlighted the synergistic impact of nutrition and muscle mass on surgical outcomes. However, given the absence of direct sarcopenia assessment in our cohort, these interpretations should be considered exploratory and serve as a basis for future prospective research.

In recent decades, there has been important progress in radiotherapy (RT) for cancer treatment [30]. RT is effective for pain reduction and local tumor control. Recent studies showed that sarcopenia or malnutrition did not seem to affect treatment outcomes and complications after radiotherapy in bladder cancer patients [31,32]. Therefore, RT may be a more appropriate option, especially in patients with poor prognostic predictors, such as a low PNI.

Our findings on survival were also consistent with previous studies. Iinuma et al. have proposed a PNI cutoff of ≥42.5 as a significant predictor of 6-month survival, closely matching the mean value in our cohort [33]. This reinforces the use of the PNI as an objective, prognostically relevant preoperative marker. Ramos et al. have compared six nutritional biomarkers—the PNI, NRI, Controlling Nutritional Status Score (CONUT), TPA, BMI, and body weight—for predicting mortality and wound complications after MST surgery [34]. They found that the PNI, NRI, and CONUT had the highest discriminatory power for 90-day and 12-month mortality (c-statistics: 0.74–0.75) and that each biomarker predicted survival independently of performance status and tumor type. Interestingly, commonly used biomarkers BMI and TPA showed lower predictive accuracies, emphasizing the superior prognostic value of composite indices such as the PNI.

Although we did not find a significant association between the PNI and postoperative infections in our cohort, it is important to interpret this in the context of broader evidence. A possible explanation might be the relatively low overall incidence of SSI in our study population or the presence of multiple contributing surgical and patient-level risk factors beyond nutrition. However, extensive evidence supports a strong link between malnutrition and postoperative infection. For instance, Tsantes et al. have conducted a meta-analysis of 22 studies including over 175,000 patients and found that malnourished patients are more than twice as likely to develop SSI following a spinal surgery (OR 2.31; 95% CI 1.75–3.05) [35]. This study underscores the clinical importance of identifying and addressing malnutrition preoperatively to reduce preventable complications. While the PNI did not correlate with infection in our analysis, its roles in inflammatory and immune responses highlight its potential as part of a broader perioperative infection risk stratification.

Beyond statistical performance, Ramos et al. have advocated for the routine incorporation of the PNI and related markers into surgical decision making, particularly because their predictive value is comparable to, or even better than, some traditional prognostic scoring systems (e.g., Tokuhashi, Tomita, Bauer) [34]. This supports our position that preoperative nutritional status should be used not only to stratify risk but also to guide preoperative optimization. Additionally, Rigney et al. have emphasized that formal preoperative nutrition consultation can reduce wound-related complications and overall morbidity after MST surgery [36]. These findings highlight the modifiable nature of malnutrition and suggest that early nutritional interventions might yield measurable improvements in surgical outcomes.

This study has several limitations. First, the retrospective design introduced potential selection bias and limited causal interpretation. Although this study was based on prospectively collected data, unmeasured confounders such as comorbidities, psychosocial factors, and/or variations in nutrition and activity might have affected outcomes. Second, the single-center setting at a tertiary hospital might reduce generalizability to other institutions with different patient demographics or perioperative protocols. We used the mean PNI value from our cohort for analysis, which may limit the generalizability of our findings. In a previous report, a PNI cutoff of ≥42.9 was suggested as a predictor of better performance status. However, multi-institutional studies are needed to establish a standardized cutoff for the PNI in the future. Third, we used the PNI as a sole nutritional marker. While convenient and validated, it does not account for muscle mass or strength, although both muscle mass and strength are core elements of sarcopenia. We could not directly assess sarcopenia in this study, although it is likely to affect ambulatory function. Also, the reliability of the PNI could be compromised in patients with certain tumor types, such as hepatocellular carcinoma, typically associated with hypoalbuminemia [37]. Fourth, although the PNI is potentially modifiable, we did not evaluate whether nutritional interventions before surgery improved functional or survival outcomes. Fifth, our results about postoperative infection could be a type II error due to its low incidence. Previous reports showed that malnourished patients are more than twice as likely to develop SSI following spine surgery. Finally, because our cohort excluded non-ambulatory patients preoperatively, our findings might not be applicable to those with severe neurological deficits or poor performance status, nor to patients undergoing nonoperative or minimally invasive treatments.

While our study demonstrated the prognostic significance of the PNI in predicting postoperative ambulatory function and overall survival, we acknowledge that its application in routine clinical practice could be further enhanced through the development of a nomogram that integrates the PNI with other relevant clinical variables. Additionally, bioinformatic approaches could be employed to refine predictive models and identify novel biomarkers associated with outcomes in MST patients. Future studies should aim to establish such models prospectively to improve risk stratification and individualized patient counseling. Also, future research should focus on integrating nutritional and sarcopenic metrics into comprehensive prognostic models and testing whether nutritional or multimodal prehabilitation strategies can improve the outcomes of MST surgery. Given the growing body of literature, randomized trials evaluating perioperative nutritional supplementation, such as those described in recent lumbar surgery studies, might be warranted in the spine oncology population.

## 5. Conclusions

The PNI is a powerful, cost-effective biomarker that can independently predict functional recovery and the survival of patients undergoing palliative spine surgery. Its clinical utility is reinforced by growing evidence from recent studies comparing nutritional biomarkers in MST. Given its prognostic robustness and ease of use, the PNI should be integrated into standard preoperative evaluation to support risk stratification, patient counseling, and perioperative planning in this vulnerable patient population.

## Figures and Tables

**Table 1:** Baseline demographic and oncological data (divided into two groups according to mean PNI [42.8]).

	Total (*n* = 133)	102 Patients Who Could Walk Independently After Surgery		
		PNI ≥ 42.8 (*n* = 59)	PNI < 42.8 (*n* = 43)	*p*
Age (years)	61.1 ± 12.2	57.7 ± 11.9	62.8 ± 12.0	0.037 #
Male	58.6% (*n* = 78)	61.0% (*n* = 36)	53.5% (*n* = 23)	0.543 *
Body mass index (kg/m^2^)	23.0 ± 3.3	22.8 ± 3.0	22.8 ± 3.9	0.927 #
Primary cancer				
Multiple myeloma	35	13	15	0.147 ***
Lung cancer	34	15	9	
Breast cancer	13	9	2	
Prostate cancer	10	3	3	
Others	39	19	14	
Prior chemotherapy	54.1% (*n* = 72)	54.2% (*n* = 32)	44.2% (*n* = 19)	0.423 *
Prior radiotherapy at index level	12.8% (*n* = 17)	11.9% (*n* = 7)	9.3% (*n* = 4)	0.757 **
Modified Charlson comorbidity index	7.1 ±1.3	6.7 ± 1.0	7.5 ± 1.4	0.001 #
ASA classification				
II	47	28	9	0.002 ***
III	71	29	28	
IV	15	2	6	
ECOG				
0	17	10	5	0.140 ***
1	59	29	19	
2	46	18	14	
3	11	2	5	
Serum albumin level (g/dL)	3.7 ± 0.6	4.1 ± 0.4	3.3 ± 0.4	<0.001 #
Lymphocyte count (/mL)	1215 ± 590	1404 ± 479	1002 ± 435	<0.001 #
Prognostic nutritional index	42.8 ± 7.5	48.4 ± 4.0	37.3 ± 4.1	<0.001 #

Independent *t*-test for #, chi-square test for *, Fisher’s exact test for **, and linear-by-linear test for *** were used.

**Table 2:** Perioperative data (divided into two groups according to mean PNI [42.8]).

	Total (*n* = 133)	102 Patients Who Could Walk Independently After Surgery		
		PNI ≥ 42.8 (*n* = 59)	PNI < 42.8 (*n* = 43)	*p*
Surgery type				
With instrumentation	89.5% (*n* = 119)	84.7% (*n* = 50)	97.7% (*n* = 42)	0.042 *
Without instrumentation	10.5% (*n* = 14)	15.3% (*n* = 9)	2.3% (*n* = 1)	
Number of instrumented levels	3.8 ± 1.7	3.6 ± 1.7	3.5 ± 1.5	0.665 #
Blood loss (mL)	906.6 ± 815.9	773.3 ± 809.2	1046.8 ± 901.4	0.118 #
ICU care	33.1% (*n* = 44)	32.2% (*n* = 19)	23.3% (*n* = 10)	0.323 *

Independent *t*-test for # and chi-square test for * were used.

**Table 3:** Relationship between the Prognostic Nutritional Index (PNI) and the occurrence of infectious conditions.

	Surgical Site Infections		Other Infections	
PNI value	Yes (*n* = 10)	No (*n* = 123)	Yes (*n* = 13)	No (*n* = 120)
	39.5 ± 6.2	43.1 ± 7.6	41.1 ± 11.5	43.0 ± 7.0
*p*	0.148		0.568	

Independent *t*-test was used.

## Data Availability

Original data will be made available upon reasonable request.

## Abbreviations

The following abbreviations are used in this manuscript:

MST	Metastatic spine tumor
NRI	Nutritional risk index
PNI	Prognostic nutritional index
SSI	Surgical site infection
BMI	Body mass index
ASA	American Society of Anesthesiologists
ECOG	Eastern Cooperative Oncology Group
mCCI	Modified Charlson Comorbidity Index
EBL	Estimated blood loss
ICU	Intensive care unit
TPA	Total psoas area
